# Changes in fatty acids during storage of artisanal‐processed freshwater sardines (*Rastrineobola argentea*)

**DOI:** 10.1002/fsn3.3284

**Published:** 2023-03-09

**Authors:** Davis Chaula, Charlotte Jacobsen, Henry S. Laswai, Bernard Elias Chove, Anders Dalsgaard, Robinson Mdegela, Grethe Hyldig

**Affiliations:** ^1^ Department of Food Sciences and Agro‐Processing Sokoine University of Agriculture Morogoro Tanzania; ^2^ National Food Institute, Division for Food Technology Technical University of Denmark Lyngby Denmark; ^3^ Faculty of Health and Medical Sciences, Section of Food Safety and Zoonoses, Department of Veterinary and Animal Sciences University of Copenhagen Frederiksberg C Denmark; ^4^ College of Veterinary and Medical Sciences, Department of Veterinary Medicine and Public Health Sokoine University of Agriculture Morogoro Tanzania

**Keywords:** fatty acid profile, Lake Victoria, omega‐3 fatty acids, *Rastrineobola argentea*

## Abstract

For ages, indigenous small fish species have been important in food and nutritional security of poor communities in low income countries. Freshwater fish, in particular fatty fish species are attracting a great attention because they are good sources of health promoting long chain omega‐3 fatty acids. Docosahexaenoic acid (DHA, C22:6*n*‐3), Docosapentaenoic acid (DPA, C22:5*n*‐3) and eicosapentaenoic acid (EPA, C20:5*n*‐3) are the main omega‐3 PUFAs known to confer health benefits in humans if consumed in required amounts. While nutritionally valued, omega‐3 PUFAs in fish are susceptible to oxidative damage during processing, transportation and subsequent storage. Lake Victoria sardines (*Rastrineobola argentea*), are rich source of chemically unstable omega‐3 fatty acids DHA, DPA and EPA. Traditionally, sardines are preserved by sun drying, deep frying and smoking. Sardine products are transported, stored and marketed at ambient temperatures. Generally, uncontrolled and higher temperatures are known to increase vulnerability of polyunsaturated fatty acids to oxidation which in turn results into loss of nutritional and sensory qualities. This study investigated changes of fat acids in sun dried, deep fried and smoked sardines during storage. Lipolysis and the progressive hydroperoxides formation were monitored by free fatty acids (FFAs) and peroxide value (PV) respectively. None volatile secondary products of lipid oxidation were measured by thiobabituric acid reactive substances (TBARS). Fatty acids were analyzed by gas chromatography with a flameionization detector (GC‐FID). Deep fried sardines maintained the lowest and apparently stable PV, TBARS and FFAs. Proportions of saturated fatty acids and polyunsaturated fatty acids decreased with time while that of monounsaturated fatty acids increased. Omega‐3 fatty acids EPA, DPA and DHA decreased with increase in storage time. In 21 days of storage, DHA was oxidized beyond detectable levels in all sardine products. Gradual increase in FFAs in sun dried sardines was suggestive of lipid hydrolysis induced by enzymes.

## Graphical abstract

Lake Victoria sardines are rich in nutritionally valued but chemically unstable omega‐3 fatty acids. Proportions of polyunsaturated fatty acids decrease with storage time. Investigations on methods to improve oxidative stability of whole sardine products during storage are needed in future studies.

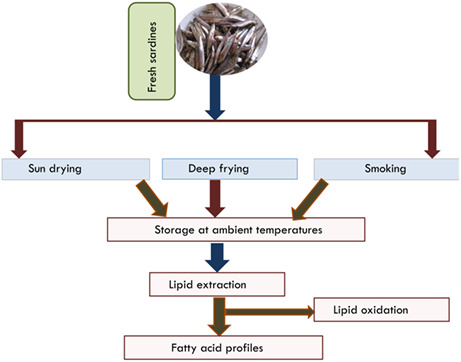

## 1 INTRODUCTION

Recently, fatty fish species have attracted researchers' attention in the search for good sources of omega‐3 polyunsaturated fatty acids (PUFAs). The three important omega‐3 fatty acids, docosahexaenoic acid (DHA), docosapentaenoic acid (DPA), and eicosapentaenoic acid (EPA), have been shown to have health benefits because of their involvement in reducing the incidence of heart attacks and inflammation and prevention of cancer cells development and brain development in fetuses (Calder, 2011; Cunnane & Stewart, 2010; Minihane et al., 2008; Sidhu, 2003; Terry et al., 2001).

Due to increased evidence for their health benefits, the best possible dietary intake of total DHA and EPA above 250 mg per day has been recommended (WHO, 2003; EFSA, 2010). In addition, PUFAs can be acquired through the dietary intake of fish and other marine food sources reported containing relatively large amounts of EPA and DHA (Cunnane & Stewart, 2010; Kuipers et al., 2010).

Polyunsaturated fatty acids show a soaring propensity to oxidation, leading to decreased nutritional value and qualitative decay of fish. This is associated with unpleasant sensory properties, commonly described as rancidity (Jacobsen et al., 1999). In addition to microbial degradation, lipid oxidation reactions are responsible for the limited shelf stability of fish products. Therefore, it is important that the handling, processing, and storage of fish products aim to minimize oxidative damage of PUFAs.

Sardines (*Rastrineobola argentea*) are known as “*dagaa*” in Tanzania. It is one of the most important commercial fish species of Lake Victoria. The small indigenous fish species constitute 75% of the total catches by weight on the Tanzanian side of the Lake (United Republic of Tanzania, 2014). The small, fatty silvery “*dagaa”* are known to be a good source of PUFAs; vitamins A, D, and the vitamin B family; minerals calcium, iron, zinc, copper, and iodine; and high‐quality protein (Chaula et al., 2019; Kabahenda et al., 2011; Owaga et al., 2010; Robert et al., 2014). Small indigenous fish species play a significant role in the food and nutrition security of economically marginalized communities in low‐income countries (Belton & Thilsted, 2014; Gurung, 2016; Hanif et al., 2016; Nanna et al., 2007).

Freshly harvested sardines are highly perishable. Thus, sun drying, smoking, and salting, the traditional fish preservation methods (Bellagha et al., 2007), have been employed to extend shelf life. Open sun drying is the main widespread low‐technology used to process Lake Victoria sardines. Other processing and preservation methods include deep frying, refrigerating, and freezing. Storage conditions of processed sardines play a central role in nutritional and sensory quality maintenance. Traditionally, sardines are processed, transported, stored, and marketed at ambient temperatures. Uncontrolled high storage temperatures are known to increase the vulnerability of polyunsaturated fatty acids to oxidative reactions (Atayeter & Ercoşkun, 2011).

The present work investigated lipid oxidation and the associated changes in fatty acids during sun‐dried, smoked, and deep‐fried sardines storage at ambient temperatures. Information obtained in this study will form the basis for finding possibilities to stabilize lipids against oxidation during the processing, transport, storage, and marketing of sardine products.

## 2 MATERIALS AND METHODS

### 2.1 Sardine sample preparation

A total of 30 samples (15 sun‐dried, eight fried, and seven smoked) were obtained from fish processors, packed in clean polyethylene bags containing 250 g fish, and kept in an insulated box. Samples were brought to the National Food Institute, Technical University of Denmark, for experiment and analysis.

The polyethylene bags containing fish were kept in the open air at an average ambient temperature (21°C) for 21 days. The ambient temperatures during storage were monitored using a Tinytag temperature logger (Gemini Data Loggers Ltd). During storage, a 50 g portion of the fish sample was taken from each bag on days 0, 7, 14, and 21 for analyses. Fish mince was obtained by homogenizing a 50 g portion of whole fish in a food chopper (MoulinexMoulinette S type 643 02210). The fish mince was stored at −40°C awaiting chemical analysis.

### 2.2 Lipid extraction

Lipid extracts from fish mince were obtained using Bligh and Dyer method with modifications according to Iverson, Lang, and Couper (Bligh & Dyer, 1959; Iverson et al., 2001). For that, a 5 g portion of fish mince was homogenized in 1:1:0.8 v/v chloroform, methanol, and water mixture at 15,000 rpm for 90 s using an Ultra Turrax homogenizer (T25 Homogenizer) followed by centrifugation at 2800 rpm and 18°C for 10 min using a centrifuge (Sigma 4 K15).

### 2.3 Peroxide values and thiobarbituric acid‐reactive substances

The peroxide value (PV) of extracted fish lipids was determined based on forming an iron‐thiocyanate complex (Shantha & Decker, 1994). The colored complex was measured using a spectrophotometer (Shimadzu UV1800, Shimadzu Scientific Instruments) at 500 nm. The analysis was performed in duplicate, and the average values of the results were expressed in milliequivalents peroxide per kg oil (meq O_2_/kg oil).

Thiobarbituric acid‐reactive substances (TBARS) were determined by a previously reported assay using 1, 1, 3‐tetra ethoxypropane (TEP) as a standard (Salih et al., 1987). The 5 g of fish mince was homogenized in 30 ml of TCA solution (7.5% TCA, 0.1% EDTA, and 0.1% propylgallate) and filtered. Five ml of the aqueous extract was allowed to react with an equal volume of 0.02 M TBA reagent in a water bath at 90°C for 40 min. The absorbance of the pink‐colored complex was measured at 530 nm. The assay was duplicated, and results were reported in μmol malonaldehyde per kg fish (μmol MDA/kg fish).

### 2.4 Free fatty acids and fatty acid profile

Acidometric titration of the lipid extract using NaOH (0.1 M) was employed to determine free fatty acid (FFA) content. FFA content was calculated according to AOCS (AOCS, 1999), and the results were reported as % oleic acid.

The fatty acid composition in fish oils from the three sardine products was determined by gas chromatography (HP 5890A; Agilent Technologies) fitted with a flame ionization detector (GC‐FID). The oven temperature program for separation was from 160 to 200°C, then from 200 to 220°C and from 220 to 240°C at 10.6°C/min. Lipid extracts were prepared for GC analysis by derivatization of extracted total lipid to fatty acid methyl esters (FAMEs). In particular, acid (BF_3_) was catalyzed, and microwave‐assisted fatty acid methylation was used. The internal standard was 2% (W/v) C 23:0 in n‐heptane. All analyses were done in duplicate, and average results were recorded. Fatty acids were expressed as g fatty acid/100 g fish oil.

### 2.5 Statistical analysis

Data were analyzed using SPSS for Windows Version 20.0 (IBM) and reported as mean ± standard deviation. Mean values at each specified time interval during storage were discriminated against using a one‐way analysis of variance (one‐way ANOVA) with the Tukey's HSD post hoc test. A *p*‐value < .05 was considered statistically significant.

## 3 RESULTS AND DISCUSSION

### 3.1 Changes in peroxide values

**FIGURE 1:**
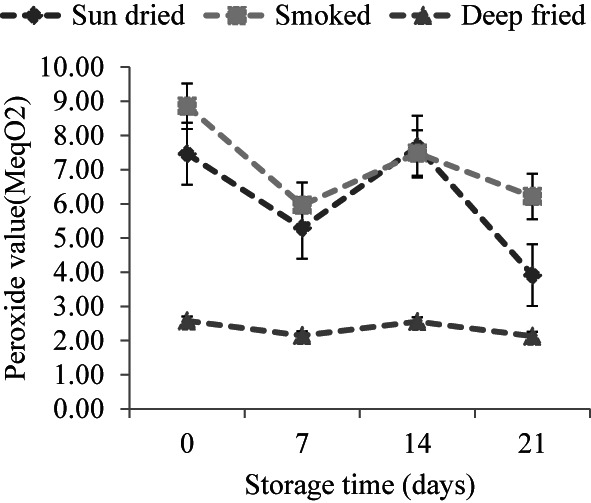
Peroxide values (PV) in sun‐dried, smoked, and deep‐fried sardines for a 21‐day storage period at ambient temperatures (21 ± 0.71°C). Results are expressed as mean ± standard deviation (*n* = 15, 8, 7 for sun‐dried, deep‐fried, and smoked products, respectively).

Lipid hydroperoxides were quantified and expressed, as shown in Figure 1. Between day 0 and day 7, a significant decrease in peroxide value was observed for sun‐dried and smoked fish. Similar trends have been noticed in fish oil‐fortified nutritional bars, fish cakes enriched with long‐chain omega‐3 fatty acids, and granola bars enriched with fish oil emulsions (Hughes et al., 2012; Dellarosa et al., 2015; Hermund et al., 2016). Peroxide value increased significantly up to day 14, followed by a decrease in the last 7 days. There were significant differences in PV among deep‐fried, sun‐dried, and smoked fish, indicating that both storage time and processing method impact the quality of lipids. Fried fish showed rather constant values of lipid hydroperoxides, about 2.3 meq O_2_ per kg of lipid, throughout 21 days of storage. PV's rise and fall patterns in sun‐dried and smoked samples were presumed to be due to the formation and decomposition of hydroperoxides. A similar trend was observed by Guizani et al. (2014) for smoked tuna during storage at 4°C. Hydroperoxides break down in several steps, yielding various rancid secondary products of lipid oxidation, including aldehydes. Results show that peroxidation was more pronounced in sun‐dried and smoked sardines during storage. This is because the products were initially found to contain a relatively high amount of long‐chain polyunsaturated fatty acids (Table 1). Fish oil extracts from these products had high proportions of omega‐3 fatty acids, making them prone to oxidation (Secci et al., 2016; Tengku‐Rozaina & Birch, 2013). Omega‐3 fatty acids have many double bonds and bisallylic carbons with low activation energy favoring hydrogen loss, free radicals, and hydroperoxide formation (Shahidi & Zhong, 2010).

**TABLE 1:** Changes in fatty acid profiles of sun‐dried, smoked, and deep‐fried sardines for a 21‐day storage period at ambient temperatures.

Product	Sun‐dried				Smoked				Deep‐fried			
Time (days)	0	7	14	21	0	7	14	21	0	7	14	21
Fatty acids (g/100 g oil)*												
14:0	3.35 ± 0.28	3.67 ± 0.05	3.41 ± 0.31	3.49 ± 1.09	1.91 ± 0.21	3.17 ± 0.42	2.94 ± 0.12	2.86 ± 0.05	0.54 ± 0.05	1.83 ± 0.12	1.75 ± 0.08	1.64 ± 0.05
15:0	0.62 ± 0.07	0.65 ± 0.01	0.62 ± 0.04	0.64 ± 0.24	0.49 ± 0.01	0.46 ± 0.05	0.43 ± 0.01	0.42 ± 0.01	0.21 ± 0.03	0.24 ± 0.03	0.21 ± 0.01	0.20 ± 0.01
16:0	21.72 ± 1.20	23.01 ± 0.57	16.24 ± 0.09	9.35 ± 0.24	21.84 ± 0.2	20.54 ± 2.58	18.95 ± 0.42	18.71 ± 0.19	28.72 ± 4.2	33.44 ± 1.89	34.69 ± 0.62	32.48 ± 0.02
17:0	0.73 ± 0.21	0.14 ± 0.01	0.18 ± 0.02	0.14 ± 0.05	0.24 ± 0.10	0.21 ± 0.02	0.19 ± 0.01	0.19 ± 0.01	0.11 ± 0.08	0.07 ± 0.01	0.04 ± 0.02	0.04 ± 0.001
18:0	7.20 ± 0.64	7.13 ± 0.16	2.54 ± 0.48	0.06 ± 0.01	4.00 ± 0.54	7.09 ± 0.86	ND	ND	0.87 ± 0.12	2.92 ± 0.41	ND	0.11 ± 0.02
20:0	0.33 ± 0.10	0.38 ± 0.01	0.26 ± 0.03	ND	0.15 ± 0.08	0.29 ± 0.03	0.27 ± 0.02	ND	0.32 ± 0.01	0.38 ± 0.07	0.38 ± 0.03	0.01 ± 0.002
Total SFAs	33.95^a^ ± 8.29	34.98^a^ ± 8.84	23.25^b^ ± 6.20	13.68^c^ ± 3.96	28.63^d^ ± 8.49	31.76^d^ ± 7.93	22.78 ^e^ ± 8.13	22.18 ^e^ ± 8.86	30.77^f^ ± 11.56	38.88^g^ ± 13.25	37.07^h^ ± 15.26	34.48^j^ ± 13.11
14:1	0.05 ± 0.03	0.06 ± 0.01	0.07 ± 0.01	0.07 ± 0.02	0.05 ± 0.01	0.06 ± 0.01	0.06 ± 0.01	0.06 ± 0.01	ND	ND	ND	0.02 ± 0.005
16:1 (*n*‐7)	10.87 ± 1.05	12.02 ± 0.23	15.95 ± 3.2	18.12 ± 3.9	5.92 ± 0.79	5.93 ± 0.83	5.19 ± 1.25	4.83 ± 0.75	1.02 ± 0.18	3.79 ± 0.66	3.33 ± 0.25	0.04 ± 0.001
18:1 (*n*‐9)	4.99 ± 0.31	7.01 ± 2.23	6.46 ± 0.48	7.01 ± 2.18	2.67 ± 0.08	2.94 ± 0.42	6.74 ± 0.02	6.69 ± 0.04	4.62 ± 1.01	5.36 ± 1.43	8.36 ± 1.74	ND
18:1 (*n*‐7)	2.64 ± 0.16	2.23 ± 1.77	5.89 ± 1.58	6.91 ± 1.11	0.34 ± 0.01	3.82 ± 1.60	4.75 ± 0.02	4.75 ± 0.04	4.30 ± 0.76	10.68 ± 1.80	14.65 ± 0.26	20.69 ± 4.70
20:1 (*n*‐9)	0.99 ± 0.09	0.17 ± 0.01	0.3 ± 0.08	0.37 ± 0.08	0.17 ± 0.01	0.19 ± 0.01	0.19 ± 0.01	0.19 ± 0.01	0.11 ± 0.05	0.06 ± 0.02	0.056 ± 0.01	0.28 ± 0.09
22:1 (*n*‐11)	0.08 ± 0.01	0.01 ± 0.02	0.22 ± 0.02	0.29 ± 0.06	ND	0.18 ± 0.02	0.25 ± 0.04	0.23 ± 0.01	ND	ND	ND	0.16 ± 0.01
22:1 (*n*‐9)	0.06 ± 0.03	0.05 ± 0.04	0.04 ± 0.01	0.04 ± 0.01	0.02 ± 0.01	0.03 ± 0.004	ND	ND	ND	ND	ND	0.01 ± 0.001
24:1 (*n*‐9)	0.86 ± 0.3	0.26 ± 0.03	0.33 ± 0.02	0.48 ± 0.03	0.36 ± 0.07	0.53 ± 0.08	0.59 ± 0.05	0.73 ± 0.22	0.2 ± 0.04	0.08 ± 0.01	0.07 ± 0.01	0.87 ± 0.08
Total MUFAs	20.54^a^ ± 3.76	21.81^b^ ± 4.46	29.26^c^ ± 5.66	33.29^d^ ± 6.40	9.53 ^e^ ± 2.22	13.68^f^ ± 2.25	17.77^g^ ± 2.89	17.48^g^ ± 2.82	10.25^h^ ± 2.23	19.97^i^ ± 4.39	26.46^j^ ± 6.24	22.07^k^ ± 7.74
16:4 (*n*‐3)	0.53 ± 0.2	0.18 ± 0.02	0.23 ± 0.02	0.24 ± 0.01	0.29 ± 0.15	0.22 ± 0.03	0.19 ± 0.01	0.19 ± 0.01	0.12 ± 0.03	0.06 ± 0.01	0.06 ± 0.01	0.12 ± 0.01
18:3 (*n*‐3)	0.32 ± 0.15	0.47 ± 0.01	0.18 ± 0.03	0.05 ± 0.01	0.27 ± 0.08	0.23 ± 0.02	0.05 ± 0.002	0.05 ± 0.01	0.10 ± 0.04	0.14 ± 0.08	0.074 ± 0.01	0.05 ± 0.003
18:4 (*n*‐3)	0.03 ± 0.03	0.09 ± 0.01	0.3 ± 0.01	0.45 ± 0.01	0.02 ± 0.02	0.21 ± 0.01	0.31 ± 0.04	0.29 ± 0.04	0.01 ± 0.01	0.12 ± 0.08	0.14 ± 0.01	0.09 ± 0.007
20:3 (*n*‐3)	ND	ND	0.17 ± 0.02	0.27 ± 0.07	0.28 ± 0.03	0.13 ± 0.08	0.28 ± 0.01	0.26 ± 0.01	0.12 ± 0.08	ND	ND	0.07 ± 0.006
20:4 (*n*‐3)	0.51 ± 0.02	0.37 ± 0.13	0.35 ± 0.05	0.42 ± 0.14	3.59 ± 1.3	0.54 ± 0.07	0.49 ± 0.02	0.46 ± 0.01	1.67 ± 1.01	0.08 ± 0.01	0.16 ± 0.01	0.15 ± 0.01
20:5 (*n*‐3) EPA	6.76 ± 0.14	6.03 ± 0.87	6.17 ± 0.52	5.6 ± 1.05	6.12 ± 1.03	5.59 ± 0.75	5.46 ± 0.03	3.25 ± 0.46	2.52 ± 0.63	2.01 ± 0.72	1.93 ± 0.22	0.53 ± 0.11
21:5 (*n*‐3)	0.34 ± 0.11	0.25 ± 0.01	0.29 ± 0.02	0.29 ± 0.12	0.33 ± 0.01	0.32 ± 0.04	0.29 ± 0.02	0.28 ± 0.01	0.11 ± 0.03	0.09 ± 0.02	0.09 ± 0.01	0.09 ± 0.002
22:5 (*n*‐3) DPA	2.01 ± 0.05	1.84 ± 0.26	1.53 ± 0.15	0.62 ± 0.01	2.27 ± 0.56	2.46 ± 0.01	1.05 ± 0.42	1.95 ± 0.01	0.88 ± 0.25	0.75 ± 0.22	0.61 ± 0.06	0.37 ± 0.03
22:6 (*n*‐3) DHA	9.04 ± 2.37	5.19 ± 0.21	0.24 ± 0.24	ND	12.36 ± 0.44	5.57 ± 0.19	0.14 ± 0.01	ND	4.94 ± 1.18	2.21 ± 0.34	0.33 ± 0.26	ND
Total *n*‐3	19.54^a^ ± 3.47	14.42^b^ ± 2.42	9.46^c^ ± 1.97	7.94^d^ ± 1.87	25.53 ^e^ ± 4.13	15.27^f^ ± 2.32	8.26^g^ ± 1.73	6.73^h^ ± 1.15	10.47^i^ ± 1.67	5.46^j^ ± 0.91	3.39^k^ ± 0.64	1.47^L^ ± 0.17
16:2 (*n*‐4)	1.44 ± 0.44	0.79 ± 0.05	0.73 ± 0.09	0.95 ± 0.24	0.98 ± 0.07	0.98 ± 0.01	0.93 ± 0.06	0.83 ± 0.34	0.29 ± 0.01	0.34 ± 0.11	0.21 ± 0.07	0.19 ± 0.07
18:2 (*n*‐4)	1.61 ± 0.70	0.26 ± 0.03	0.17 ± 0.07	0.16 ± 0.11	0.23 ± 0.04	0.24 ± 0.16	0.11 ± 0.06	0.09 ± 0.01	0.22 ± 0.01	0.15 ± 0.01	0.13 ± 0.05	0.06 ± 0.002
16:3 (*n*‐4)	0.43 ± 0.06	0.15 ± 0.01	1.12 ± 0.06	1.66 ± 0.31	1.36 ± 0.12	0.72 ± 0.09	1.41 ± 0.04	0.06 ± 0.01	0.56 ± 0.02	0.05 ± 0.01	0.05 ± 0.01	0.02 ± 0.01
18:3 (*n*‐4)	1.97 ± 0.57	1.02 ± 0.04	0.31 ± 0.01	0.29 ± 0.06	1.84 ± 0.05	0.34 ± 0.10	0.22 ± 0.16	0.11 ± 0.01	0.47 ± 0.03	0.11 ± 0.06	0.14 ± 0.02	0.11 ± 0.04
Total *n*‐4	5.45 ± 0.66	2.22 ± 0.42	2.33 ± 0.43	3.06 ± 0.69	4.41 ± 0.68	2.28 ± 0.34	2.67 ± 0.61	1.09 ± 0.37	1.54 ± 0.16	0.65 ± 0.13	0.53 ± 0.07	0.38 ± 0.07
18:2 (*n*‐6)	2.13 ± 0.65	2.33 ± 0.02	0.68 ± 0.11	0.09 ± 0.01	0.85 ± 0.1	0.85 ± 0.11	0.07 ± 0.03	0.06 ± 0.01	6.04 ± 0.5	3.90 ± 0.50	3.58 ± 0.50	0.11 ± 0.06
20:2 (*n*‐6)	0.17 ± 0.04	0.28 ± 0.05	0.1 ± 0.06	0.08 ± 0.01	0.17 ± 0.01	0.13 ± 0.02	ND	ND	0.11 ± 0.01	0.09 ± 0.01	0.06 ± 0.01	0.01 ± 0.05
18:3 (*n*‐6)	1.02 ± 0.09	0.26 ± 0.04	0.65 ± 0.01	0.26 ± 0.08	0.29 ± 0.06	1.11 ± 0.13	0.24 ± 0.01	0.17 ± 0.01	0.14 ± 0.01	0.09 ± 0.01	0.08 ± 0.01	0.09 ± 0.01
20:3 (*n*‐6)	2.64 ± 0.93	2.52 ± 0.25	0.93 ± 0.07	ND	2.58 ± 0.14	1.25 ± 0.17	ND	ND	1.15 ± 0.05	0.94 ± 0.23	0.82 ± 0.13	0.04 ± 0.005
20:4 (*n*‐6)	0.26 ± 0.01	0.29 ± 0.03	0.09 ± 0.01	0.06 ± 0.01	0.32 ± 0.02	0.19 ± 0.02	0.05 ± 0.001	0.05 ± 0.001	0.09 ± 0.003	0.10 ± 0.02	0.08 ± 0.008	0.01 ± 0.001
Total *n*‐6	6.22 ± 1.11	5.68 ± 1.18	2.45 ± 0.38	0.49 ± 0.09	4.21 ± 1.01	3.53 ± 0.52	0.36 ± 0.10	0.28 ± 0.07	7.53 ± 2.57	5.12 ± 1.65	4.62 ± 1.52	0.26 ± 0.05
Total PUFAs	31.21^a^ ± 2.45	22.32^b^ ± 1.79	14.24^c^ ± 1.39	11.49^d^ ± 1.36	34.15 ^e^ ± 3.05	21.08^f^ ± 1.70	11.29^c^ ± 1.32	8.10^h^ ± 0.89	19.54^i^ ± 1.74	11.23^j^ ± 1.07	8.54^k^ ± 0.92	2.11^L^ ± 0.13

*Abbreviations*: SFA, saturated fatty acids; MUFA, monounsaturated fatty acids; PUFA, polyunsaturated fatty acids; ND, not detected.

* Fish lipid was extracted from whole fish body. Values are expressed in mean ± standard deviation (*n* = 15 for sun‐dried, *n* = 7 for smoked, and *n* = 8 for deep‐fried sardines). Values with different letters in a row for each product are statistically significant.

Hydroperoxides are a quality marker for products containing lipids, mainly when lipid quality defines a product's shelf stability. However, the use of peroxide value as the sole index of lipid quality could be misleading due to their high instability.

### 3.2 Changes in TBARS

**FIGURE 2:**
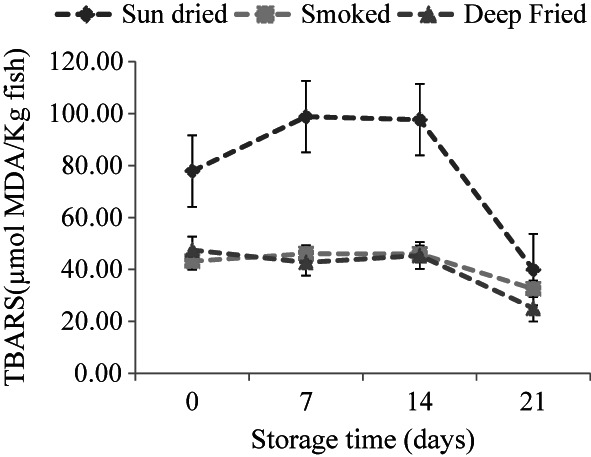
Thiobarbituric acid‐reactive substances (TBARS) in sun‐dried (*n* = 15), smoked (*n* = 7), and deep‐fried sardines (*n* = 8) for a 21‐day storage period at ambient temperatures (21 ± 0.71°C). Results are expressed as mean ± standard deviation.

**FIGURE 3:**
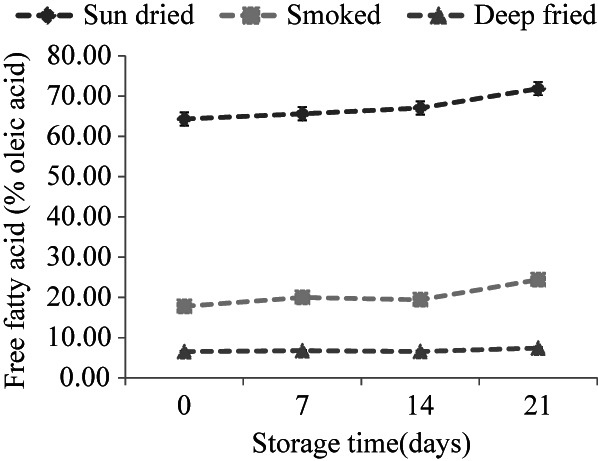
Free fatty acids in sun‐dried (*n* = 15), smoked (*n* = 7), and deep‐fried sardines (*n* = 8) for a 21‐day storage period at ambient temperatures (21 ± 0.71°C). Results are expressed as mean ± standard deviation.

Thiobabituric acid reactive substances assay measures the content of the nonvolatile secondary lipid degradation product, malonaldehyde, derived from the decomposition of the lipid hydroperoxides. Changes in TBARS in sardines during storage are shown in Figure 3. TBARS values in smoked and fried sardines were significantly lower than in sun‐dried sardines. Thus, secondary oxidation was greater in sun‐dried samples compared with smoked and deep‐fried sardines. However, TBARS in sun‐dried sardines increased significantly between day 0 and day 7 of storage and remained unchanged between day 7 and 14. A decrease in TBARS was noticed (*p* < .05) between day 14 and day 21. Guizani et al., 2014 reported a similar trend in the content of TBARS for salted and smoked tuna stored at 4°C for 49 days. Increased and decreased TBARS in fish oil‐enriched pâté were also reported during 12 weeks of storage at 2–10°C (Nielsen & Charlotte, 2013). The highest TBARS values, equivalent to 7.12 and 7.04 mg malonaldehyde/kg fish, were obtained for sun‐dried samples on days 7 and 14 of storage, respectively. These values are above 6 mg malonaldehyde/kg fish, which is acceptable for developing an objectionable odor in fish products (Freeman & Hearnsberger, 1994). Due to the high proportions of polyunsaturated fatty acids in sun‐dried sardines, oxidation could occur rapidly during the first 7 days of storage. The marked increase in TBARS in sun‐dried samples between day 0 and day 7 of storage coincided with a decrease in their peroxide value (Figures 1 and 2). This was probably due to the decomposition of formed hydroperoxides into the secondary oxidation products. In many cases, hydroperoxides can be oxidized to ketones and malonaldehyde in the later stages of lipid oxidation. Chaijan et al. (2006) reported a marked increase in TBARS throughout the 15 days of iced storage of sardine (*Sardinella gibbosa*). Although increased and decreased peroxide values were observed in smoked sardines, the corresponding TBARS values remained low and rather constant throughout the 21 days of storage time. This could be due to the antioxidative effect of smoking, which results from the combination of dehydration and smoke constituents (Goulas & Kontominas, 2005; Guillen & Errecalde, 2002). Although the oxidative characteristics of frying oil were not analyzed in this study, nonvolatile secondary products of lipid oxidation are reported to accumulate in oil during batch frying (Urbancic et al., 2014). Partly, this might account for the lower TBARS value in deep‐fried samples.

### 3.3 Changes in free fatty acids

Changes in free fatty acids (FFAs) in sardines during storage are depicted in Figure 3. FFAs remained lower and more stable in smoked and deep‐fried than in sun‐dried sardines. This observation indicates that lipid hydrolysis occurred to a greater extent in sun‐dried sardines. During analysis, sun‐dried sardines had higher moisture content than fried and smoked fish (results not presented), which might have favored enzyme activity. In another study, it was found that FFAs in sun‐dried sardines were correlated (*r* = 0.543) with moisture content (Chaula et al., 2019). Hydrolysis of glycerol fatty acid esters occurs in fish lipids with the liberation of free fatty acids whose accumulation has been associated with unpleasant sensory properties (Lopez‐Amaya & Marangoni, 2000). Specifically, the main enzymes involved in fish lipid hydrolysis are triacyl lipase, phospholipase A2, and phospholipase B (Shah et al., 2009). The enzymes are found in fish and could also be produced by certain microorganisms present in dried sardines (Zebedayo et al., 2017), therein contributing to the lipolytic breakdown of fish lipids. High temperatures could have inactivated these enzymes during deep frying and smoking of sardines resulting in low and stable FFAs content in the products throughout storage time.

### 3.4 Changes in fatty acid profile

Fatty acids in the three processed sardines products were determined at specified time intervals, and the amount of each expressed in g fatty acid/100 g fish oil is presented in Table 1. Amounts of saturated fatty acids (SFAs), monounsaturated fatty acids (MUFAs), and polyunsaturated fatty acids (PUFAs) changed differently among the three types of products during storage. SFAs clearly represented the major group ranging from 13.7 to 38.9% of total lipid content. During the entire storage time of 21 days, the ranges of SFA, MUFAs, and PUFAs were different among the three sardine products. Sun‐dried sardine comprised 13.7–34.9% SFAs, 20.5–33.35% MUFAs, and 11.5–31.2% PUFAs, whereas smoked sardine consisted of 22.2–31.8% SFAs, 9.5–17.8% MUFAs, and 8.1–34.2% PUFAs and deep‐fried sardines contained 30.8–38.9% SFAs, 10.3–26.5% MUFAs, and 2.1–19.5% PUFAs.

Palmitic acid was the dominant SFA in all samples, with deep‐fried containing up to 34.5% of the total lipid content. The high amount of palmitic acid in deep‐fried samples is presumably because palm oil was used to deep‐fry the product. The proportions of MUFAs in all samples increased with time, whereas the total PUFAs decreased (Table 1). The major PUFAs were eicosapentaenoic acid (EPA), docosapentaenoic acid (DPA), and docosahexaenoic acid (DHA). Generally, lipids in deep‐fried sardines contained the lowest proportions of PUFAs suggesting their damage due to high temperature during frying processing. A decrease in PUFAs with time in all samples and changes in PV and TBARS indicate that lipid oxidation transpired at varying rates in different sardine products during storage. Correspondingly, there was a decrease in the nutritionally valued omega‐3 fatty acids during storage. After 10 days of storage, a significant decrease in the total amount of PUFAs was seen in dried herring fillets (Shah et al., 2009).

DHA was the most abundant PUFA, followed by EPA and DPA in all samples. After 7 days of storage, DHA decreased by 42.6%, 54.9%, and 55.3%, whereas EPA decreased by 10.8%, 8.7%, and 20.2% in sun‐dried, smoked, and deep‐fried sardines, respectively. After 14 days of storage, DHA decreased by 97.3%, 98.9%, and 93.3%, whereby EPA decreased by 8.7%, 10.8%, and 23.4% in sun‐dried, smoked, and deep‐fried sardines, respectively. Chaijan et al., 2006 reported that the storage of sardines (*Sardinella gibbosa*) for 6 days under ice induced 13.2 and 5.5% decreases in EPA and DHA, respectively. The marked decrease in DHA and EPA might be due to their vulnerability to oxidation. Results in this study show that after 21 days of storage, DHA was not detected in any sample, indicating that the lipid oxidation reaction had exhausted the available DHA beyond the detection limit. Generally, the susceptibility of lipids to peroxidation in food depends on the lipid composition, the presence of pro‐oxidants and antioxidants, oxygen levels, temperature, light, and processing methods (Kolakowska & Bartosz, 2014).

## 4 CONCLUSIONS

Storage of sardines at ambient temperatures is associated with large changes in fatty acid composition simultaneously with progressive lipid oxidation and hydrolysis. It was found that the proportions of DHA, EPA, and DPA decreased significantly during the storage period indicating a loss of nutritional and sensory quality. These results suggest that the oxidation of PUFAs would lead to the formation of degradation products, off‐flavors, and other sensory alterations, reducing product acceptability, quality, and shelf stability. Investigations on sardine consumption patterns, expected intake of omega‐3 fatty acids, and methods to improve the oxidative stability of sardine products during storage are needed in future studies.

## CONFLICT OF INTEREST

The authors have no conflict of interest to declare.

## Data Availability

The data that support findings of this study are available from the corresponding author upon request.

## References

[fsn33284-bib-0001] AOCS . (1999). AOCS official method Ca 5a‐40: Free fatty acids. In Official methods and recommended practices of the American oil Chemists Society. AOCS.

[fsn33284-bib-0002] Atayeter, S. , & Ercoşkun, H. (2011). Chemical composition of European squid and effects of different frozen storage temperatures on oxidative stability and fatty acid composition. Journal of Food Science and Technology, 48(1), 83–89. 23572720 10.1007/s13197-010-0139-5 PMC3551084

[fsn33284-bib-0003] Bellagha, S. , Sahli, A. , Farhat, A. , Kechaou, N. , & Glenza, A. (2007). Studies on salting and drying of sardine (*Sardinella aurita*): Experimental kinetics and modeling. Journal of Food Engineering, 78(3), 947–952.

[fsn33284-bib-0004] Belton, B. , & Thilsted, S. H. (2014). Fisheries in transition: Food and nutrition security implications for the global south. Global Food Security, 3, 59–66.

[fsn33284-bib-0005] Bligh, E. G. , & Dyer, W. J. (1959). A rapid method of total lipid extraction and purification. Canadian Journal of Biochemistry and Physiology, 37(8), 911–917. 13671378 10.1139/o59-099

[fsn33284-bib-0006] Calder, P. C. (2011). Fatty acids and inflammation: The cutting edge between food and pharma. European Journal of Pharmacology, 668(1), 50–58. 10.1016/j.ejphar.2011.05.085 21816146

[fsn33284-bib-0007] Chaijan, M. , Benjakul, S. , Visessanguan, W. , & Faustman, C. (2006). Changes of lipids in sardine (*Sardinella gibbosa*) muscle during iced storage. Food Chemistry, 99, 83–91.

[fsn33284-bib-0008] Chaula, D. , Laswai, L. , Chove, B. , Dalsgaard, A. , Mdegela, R. , & Hyldig, G. (2019). Fatty acid profiles and lipid oxidation status of sun dried, deep fried and smoked sardine (*Rastrineobola argentea*) from Lake Victoria, Tanzania. Journal of Aquatic Food Product Technology, 28(2), 165–176.

[fsn33284-bib-0009] Cunnane, S. C. , & Stewart, K. M. (2010). Human brain evolution: A new wetlands scenario. In S. C. Cunnane & K. M. Stewart (Eds.), Human brain evolution: The influence of freshwater and marine food resources (pp. 203–207). John Wiley & Sons, Inc.

[fsn33284-bib-0010] Dellarosa, N. , Luca, L. , Emilía, M. , Rosa, J. , & Kolbrún, S. (2015). Enrichment of convenience seafood with omega‐3 and seaweed extracts: Effect on lipid oxidation. LWT‐Food Science and Technology, 62, 746–752.

[fsn33284-bib-0011] EFSA . (2010). Scientific opinion on dietary reference values for fats, including saturated fatty acids, polyunsaturated fatty acids, monounsaturated fatty acids, trans fatty acids, and cholesterol. EFSA Journal, 8, 1461.

[fsn33284-bib-0012] Freeman, D. W. , & Hearnsberger, J. O. (1994). Rancidity in selected sites of frozen catfish fillets. Journal of Food Science, 59(1), 60–63.

[fsn33284-bib-0013] Goulas, A. E. , & Kontominas, M. G. (2005). Effect of salting and smoking method on the keeping quality of chub mackerel (*Scomber japonicus*): Biochemical and sensory attributes. Food Chemistry, 93(3), 511–520.

[fsn33284-bib-0014] Guillen, M. D. , & Errecalde, M. C. (2002). Volatile components of raw and smoked black bream (*Brama raii*) and rainbow trout (*Oncorhynchus mykiss*) studied by means of solid phase microextraction and gas chromatography/mass spectrometry. Journal of the Science of Food and Agriculture, 82, 945–952.

[fsn33284-bib-0015] Guizani, N. , Mohammad, S. R. , Mohamed, H. A. , Jamal, N. A. , & Sithara, S. (2014). Effects of brine concentration on lipid oxidation and fatty acids profile of hot smoked tuna (*Thunnus albacares*) stored at refrigerated temperature. Journal of Aquatic Food Product Technology, 51(3), 577–582. 10.1007/s13197-011-0528-4 PMC3931871 24587535

[fsn33284-bib-0016] Gurung, T. B. (2016). Role of inland fishery and aquaculture for food and nutrition security in Nepal. Agriculture and Food Security, 5, 18.

[fsn33284-bib-0017] Hanif, M. , Siddik, M. A. B. , Nahar, A. , Chaklader, M. R. , Rumpa, R. J. , Alam, M. J. , & Sultan, M. (2016). The current status of small indigenous fish species (SIS) of river Gorai, a distributary of the river Ganges, Bangladesh. Journal of Biodiversity and Endangered Species, 4, 162.

[fsn33284-bib-0018] Hermund, D. B. , Karadağ, A. , Andersen, U. , Jónsdóttir, R. , Kristinsson, H. G. , Alasalvar, C. , & Jacobsen, C. (2016). Oxidative stability of granola bars enriched with multilayered fish oil emulsion in the presence of novel brown seaweed‐based antioxidants. Journal of Agricultural and Food Chemistry, 64, 8359–8368. 27741399 10.1021/acs.jafc.6b03454

[fsn33284-bib-0019] Hughes, B. H. , Muzzy, H. M. , Laliberte, L. C. , Grenier, H. S. , Perkins, L. B. , & Skonberg, D. I. (2012). Oxidative stability and consumer acceptance of fish oil fortified nutrition bars. Journal of Food Science, 77, 329–334. 10.1111/j.1750-3841.2012.02870.x 22957916

[fsn33284-bib-0020] Iverson, S. J. , Lang, S. L. C. , & Couper, M. H. (2001). Comparison of the Bligh and Dyer and Fotch method for total lipid determination in a broard range of marine tissues. Lipids, 36, 1283–1287. 11795862 10.1007/s11745-001-0843-0

[fsn33284-bib-0021] Jacobsen, C. , Hartvigsen, K. , Lund, P. , Meyer, A. S. , Adler‐Nilsen, J. , Holstborg, J. , & Hølmer, G. (1999). Oxidation in fish oil‐enriched mayonnaise: Assessment of propyl gallate as an antioxidant by discriminant partial least squares regression analysis. European Food Research and Technology, 210(1), 13–30.

[fsn33284-bib-0022] Kabahenda, M. K. , Amega, R. , Okalany, E. , Husken, S. M. C. , & Heck, S. (2011). Protein and micronutrients composition of low‐value fish products commonly marketed in the Lake Victoria region. World Journal of Agricultural Sciences, 7(5), 521–526.

[fsn33284-bib-0023] Kolakowska, A. , & Bartosz, G. (2014). Oxidation of food components: An introduction. In G. Bartosz (Ed.), Food oxidants and antioxidants: Chemical, biological and functional properties (pp. 9–10). Taylor and Francis Group, CRC Press.

[fsn33284-bib-0024] Kuipers, R. S. , Martine, F. L. , Janneke Dijck‐Brouwer, D. A. , Boyd, E. S. , Michael, A. C. , Loren, C. , & Frits, A. J. M. (2010). Estimated macronutrient and fatty acid intakes from an east African Paleolithic diet. British Journal of Nutrition, 104, 1666–1687. 20860883 10.1017/S0007114510002679

[fsn33284-bib-0025] Lopez‐Amaya, C. , & Marangoni, A. (2000). Phospholipases. In F. N. Haard & K. B. Simpson (Eds.), Seafood enzymes (pp. 91–119). Marcel Dekker, Inc.

[fsn33284-bib-0026] Minihane, A. M. , Givens, D. I. , & Gibbs, R. A. (2008). Health benefits of organic food. In I. Givens , S. Baxter , A. M. Minihane , & E. Shaw (Eds.), Effects of the environment (pp. 19–49). CABI.

[fsn33284-bib-0027] Nanna, R. , Wahab, M. , Chamnan, C. , & Thilsted, S. (2007). The role of fish in food‐based strategies to combat vitamin a and mineral deficiencies in developing countries. The Journal of Nutrition, 137, 1106–1109. 17374688 10.1093/jn/137.4.1106

[fsn33284-bib-0028] Nielsen, N. S. , & Charlotte, J. (2013). Retardation of lipid oxidation in fish oil‐enriched fish pâté: Combination effects. Journal of Food Biochemistry, 37, 88–97.

[fsn33284-bib-0029] Owaga, E. E. , Onyango, C. A. , & Njoroge, C. (2010). Influence of washing treatments and drying temperatures on proximate composition of dagaa (*Rastrineobola argentea*). African Journal of Food, Agriculture, Nutrition and Development, 10, 2834–2844.

[fsn33284-bib-0030] Robert, A. , Mfilinge, P. , Limbu, S. M. , & Mwita, C. J. (2014). Fatty acid composition and levels of selected polyunsaturated fatty acids in four commercial important freshwater fish species from Lake Victoria, Tanzania. Journal of Lipids, 2014, 1–7. 10.1155/2014/712134 PMC4290058 25610654

[fsn33284-bib-0031] Salih, M. , Smith, D. M. , Price, J. F. , & Dawson, L. E. (1987). Modified extraction 2‐thiobarbituric acid method for measuring lipid oxidation in poultry. Poultry Science, 66(9), 1483–1488. 10.3382/ps.0661483 3684874

[fsn33284-bib-0032] Secci, G. , Borgogno, M. , Lupi, P. , Rossi, S. , Paci, G. , Mancini, S. , Bonelli, A. , & Parisi, G. (2016). Effect of mechanical separation process on lipid oxidation in European aquacultured sea bass, gilthead sea bream and rainbow trout products. Food Control, 67, 75–81.

[fsn33284-bib-0033] Shah, A. K. M. A. , Tokunaga, C. , Kurihara, H. , & Takahashi, K. (2009). Changes in lipids and their contribution to the taste of migaki‐nishin (dried herring fillet) during drying. Food Chemistry, 115(3), 1011–1018.

[fsn33284-bib-0034] Shahidi, F. , & Zhong, Y. (2010). Lipid oxidation and improving the oxidative stability. Chemical Society Reviews, 39(11), 4067–4079. 20617249 10.1039/b922183m

[fsn33284-bib-0035] Shantha, N. C. , & Decker, E. A. (1994). Rapid, sensitive, iron‐based spectrophotometric methods for determination of peroxide values of food. Lipids Journal of AOAC International, 77, 421–424. 8199478

[fsn33284-bib-0036] Sidhu, K. S. (2003). Health benefits and potential risks related to consumption of fish or fish oil. Regulatory Toxicology and Pharmacology, 38, 336–344. 14623484 10.1016/j.yrtph.2003.07.002

[fsn33284-bib-0037] Tengku‐Rozaina, T. M. , & Birch, E. J. (2013). Physicochemical characterization and oxidative stability of refined hoki oil, unrefined hoki oil and unrefined tuna oil. International Journal of Food Science and Technology, 48(11), 2331–2339.

[fsn33284-bib-0038] Terry, P. , Lichtenstein, P. , Feychting, M. , Ahlbom, A. , & Wolk, A. (2001). Fatty fish consumption and risk of prostate cancer. The Lancet, 357, 1764–1766. 10.1016/S0140-6736(00)04889-3 11403817

[fsn33284-bib-0039] United Republic of Tanzania . (2014). Ministry of Livestock and Fisheries development; Fisheries Development Division: Fisheries Annual report. Livestock and Fisheries development.

[fsn33284-bib-0040] Urbancic, S. , Kolar, M. H. , Dimitrijevic, D. , Demsar, L. , & Vidrih, R. (2014). Stabilisation of sunflower oil and reduction of acrylamide formation of potato with rosemary extract during deep‐fat frying. LWT ‐ Food Science and Technology, 57, 671–678.

[fsn33284-bib-0041] World Health Organization . (2003). Diet, nutrition, the prevention of chronic disease. Report of a joint WHO/FAO expert consultation. In WHO technical reports series 916. WHO.

[fsn33284-bib-0042] Zebedayo, B. , Dalsgaard, A. , Mhongole, O. J. , Madsen, H. , & Mdegela, R. H. (2017). Microbial quality and safety of fresh and dried *Rastrineobola argentea* from Lake Victoria, Tanzania. Food Control, 81, 16–22.

